# Supplementary material for: Transcriptome Analysis Reveals Vimentin-Induced Disruption of Cell–Cell Associations Augments Breast Cancer Cell Migration
Source: Cells. 2022 Dec 13;11(24):4035. doi: 10.3390/cells11244035 (PMC9776984; doi:10.3390/cells11244035)
Supplement: Supplementary file 1 [file cells-11-04035-s001.zip › DEGs and enrichment analysis.zip!/DEGs and enrichment analysis/WT_VIMvsVector KEGG/ALL/WT_VIMvsVector_ALL.pathway_map.zip!/WT_VIMvsVector_ALL.pathway_map/src/hsa04510.html]

hsa04510


- hsa:3694

- Down regulated genes

ENSG00000115221(-1.63377760371661)

- hsa:3674

- Up regulated genes

ENSG00000005961(3.91653577454454)

- hsa:824

- Down regulated genes

ENSG00000162909(-2.04343414211511)

- hsa:3918

- Down regulated genes

ENSG00000058085(-2.27781883943368)
- hsa:1285

- Down regulated genes

ENSG00000169031(-2.54975234049394)
- hsa:3915

- Down regulated genes

ENSG00000135862(-1.63927617256978)
- hsa:1287

- Down regulated genes

ENSG00000188153(-2.16664393151527)
- hsa:3909

- Down regulated genes

ENSG00000053747(-1.74718362506906)

- hsa:330

- Down regulated genes

ENSG00000023445(-2.12138419902495)

- hsa:596

- Up regulated genes

ENSG00000171791(2.03324203294485)

- hsa:3791

- Down regulated genes

ENSG00000128052(-2.65973347544558)
- hsa:2324

- Up regulated genes

ENSG00000037280(2.35155506151555)

Close
